# Mimicry of surgical site infection – Case report

**DOI:** 10.1016/j.ijscr.2020.05.034

**Published:** 2020-05-29

**Authors:** H. Bretschneider, S. Helbig, C. Kleber, K. de With, M. Stiehler

**Affiliations:** a University Centre for Orthopaedics and Trauma Surgery, University Hospital Carl Gustav Carus Dresden and Faculty of Medicine of Technische Universität Dresden, Fetscherstraße 74, 01307 Dresden, Germany; b Division of Infectious Diseases, University Hospital Carl Gustav Carus Dresden and Faculty of Medicine of Technische Universität Dresden, Fetscherstraße 74, 01307 Dresden, Germany

**Keywords:** Total joint arthroplasty, Soft tissue complications, Periprosthetic joint infection, Calciphylaxis, Case report

## Abstract

**Background context:**

Calciphylaxis is a rare complication of secondary hyperparathyroidism caused by calcifications of small blood vessels in the skin and soft tissue. The disease occurs almost exclusively in patients with chronic kidney disease and has an incidence of approximately 50 cases per year in Germany [1].

**Purpose:**

We present a case of a 61-year-old woman with calciphylaxis in connection with a primary knee endoprosthesis implantation.

**Study design:**

Case report.

**Methods:**

A review of the medical records since the time of initial hospital admission throughout the entire hospitalization until the death of the patient was performed.

**Results:**

Calciphylaxis caused severe soft tissue complications after total joint arthroplasty. Despite interdisciplinary therapy, including revision and plastic surgery as well as intensive care, the patient died 4 months after primary total knee arthroplasty due to septic multi-organ failure.

**Conclusion:**

Calciphylaxis may cause severe soft tissue complications after total joint arthroplasty and should be considered as potential differential diagnosis to surgical site infection. This is the first case report on calciphylaxis as direct complication of total joint replacement surgery.

## Highlights

• Calciphylaxis may cause severe soft tissue complications after total joint arthroplasty.
• Caliphylaxis is a potential differential diagnosis to surgical site infection in patients with chronic kidney disease.
• Skin biopsy should be taken in case of suspected calciphylaxis to confirm the diagnosis.

## 1 Introduction

Calciphylaxis is a rare syndrome caused by calcifications of small blood vessels in the skin and soft tissue, which is a complication of secondary hyperparathyroidism [2,3]. The disease occurs almost exclusively in patients with chronic kidney disease and has an incidence of approximately 50 cases per year in Germany [1]. At the beginning the patient often experiences very painful hardening of the skin, resulting in progressive and increasingly painful ulcers. Therapeutically, the aim is to reduce calcium and phosphate levels. In individual cases, surgical removal of the parathyroid glands should be considered. Infectious complications leading to sepsis can occur. Calciphylaxis precipitated by primary knee endoprosthesis implantation has not been previously described. The case report is in line with the SCARE criteria [4].

## 2 Presentation of case

**Fig. 1:**
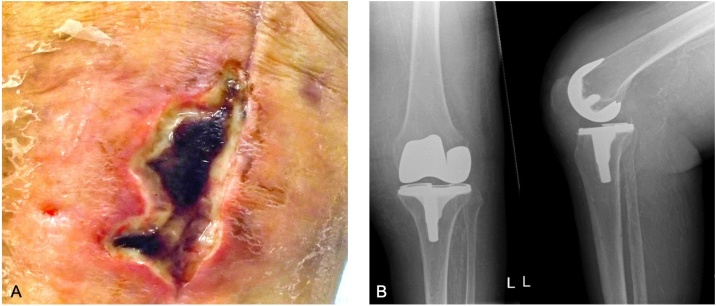
Knee of the patient with wound healing disorder (A) and x-ray images after primary total knee endoprosthesis implantation (B).

A 61-year-old woman was referred one month after primary total knee arthroplasty from a Rehabilitation Clinic to an Orthopaedic and Trauma Surgery Department of a Tertiary Care University Hospital for a presumed periprosthetic joint infection (Fig. 1). On admission, white blood cell count was normal, C-reactive protein was elevated (167 mg/dl), phosphate was reduced (0.32 mmol/l) and calcium was normal (2.36 mmol/l). The patient’s comorbidities included metabolic syndrome with type 2 diabetes mellitus accompanied by micro- and macrovascular complications. Chronic kidney disease was classified stage “G5” (kidney failure, GFR 9 mL/min, KDIGO) without haemodialysis.

Endoprosthesis explantation and implantation of a static bone cement spacer was performed and vacuum assisted closure-therapy (VAC-therapy) was applied for a prepatellar soft tissue defect. Multiple intraoperative tissue cultures grew *Enterobacter cloacae complex*. Piperacillin/tazobactam was administered according to antibiogram. Multiple VAC exchanges were performed for soft tissue conditioning. Satisfactory wound healing failed to occur despite of sterile tissue samples.

**Fig. 2:**
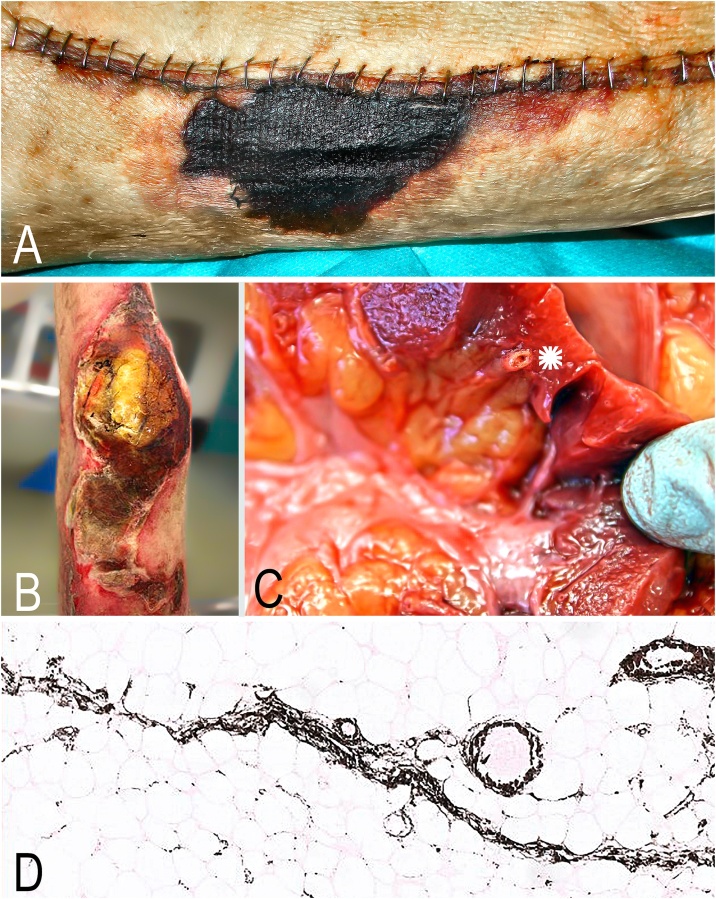
Necrosis of the edge of the wound 8 days after gastrocnemius muscular flap (A); local findings of the knee joint post mortem showing extensive skin and soft tissue necrosis (B), high-grade vascular wall sclerosis of the medium and small vessels (C*); histological verification of small vessel calcification (von Kossa staining; D).

A gastrocnemius muscular flap was applied for ongoing impaired wound healing despite consistently sterile tissue cultures. Unfortunately, flap necrosis developed after 8 days (Fig. 2A) and additional necrotic lesions occurred on the contralateral lower extremity after 15 days. A skin biopsy revealed fatty tissue necrosis with extensive interstitial calcification on histopathology. The histopathology finding in light of chronic renal disease and progressive necrotic skin lesions suggested the diagnosis of calciphylaxis. Parathyroid hormone (PTH) levels were elevated at 441 pg/mL (normal range, 11–67 pg/ml). Haemodialysis, which had been started upon worsening of renal function, was thus intensified and pharmacological parathyroid suppression was initiated by thiosulfate and cinacalcet administration. Due to persistently high PTH levels, parathyroidectomy was performed and PTH levels normalized subsequently.

## 3 Results

The patient died 4 months after primary total knee arthroplasty due to septic multi-organ failure. The autopsy findings including histopathology confirmed calciphylaxis (Fig. 2B–D).

## 4 Conclusions

Calciphylaxis may cause severe soft tissue complications after total joint arthroplasty and should be considered as potential differential diagnosis [5]. Analyzing the English literature in Pubmed, Medline, and Google this is the first case report on calciphylaxis-related complications upon total joint replacement surgery.

## Conflicts of interest

Nothing to declare.

## Funding

Nothing to declare.

## Ethical approval

This case report needs no ethnical approval.

## Consent

The names of patients and volunteers, initials or hospital numbers were not used.

Written informed consent was obtained from the patient for publication of this case report and accompanying images. A copy of the written consent is available for review by the Editor-in-Chief of this journal on request.

## Authors contribution

Conception and design: Bretschneider, Helbig, de With, Stiehler.

Analysis and interpretation of the data: all co-authors.

Writing: Bretschneider, Helbig, de With, Stiehler.

Critical revision of the article for important intellectual content: Stiehler, de With.

## Registration of research studies

Non applicable.

## Guarantor

PD Maik Stiehler.

## Provenance and peer review

Not commissioned, externally peer-reviewed.
